# Improving Hospital Care and Collaborative Communications for the 21st Century: Key Recommendations for General Internal Medicine

**DOI:** 10.2196/ijmr.2022

**Published:** 2012-09-24

**Authors:** Robert C Wu, Vivian Lo, Peter Rossos, Craig Kuziemsky, Kevin J O’Leary, Joseph A Cafazzo, Scott Reeves, Brian M Wong, Dante Morra

**Affiliations:** 1 Toronto General HospitalDivision of General Internal MedicineUniversity Health NetworkToronto, ONCanada; 2 Toronto General HospitalCentre for Innovation in Complex CareUniversity Health NetworkToronto, ONCanada; 3 Department of MedicineUniversity of TorontoToronto, ONCanada; 4 Shared Information Management ServicesUniversity of Health NetworkToronto, ONCanada; 5 Centre for Global eHealth InnovationUniversity Health NetworkToronto, ONCanada; 6 Telfer School of ManagementUniversity of OttawaOttawa, ONCanada; 7 Feinberg School of MedicineDepartment of MedicineNorthwestern UniversityChicago, ILUnited States; 8 Department of Health Policy, Management and EvaluationUniversity of TorontoToronto, ONCanada; 9 Institute of Biomaterials and Biomedical EngineeringUniversity of TorontoToronto, ONCanada; 10 Centre for Faculty DevelopmentLi Ka Shing International Health Care Education CentreSt Michael's HospitalToronto, ONCanada; 11 Li Ka Shing Knowledge InstituteKeenan Research CentreSt Michael’s HospitalToronto, ONCanada; 12 Wilson Centre for Research in EducationUniversity Health NetworkToronto, ONCanada; 13 Department of PsychiatryUniversity of TorontoToronto, ONCanada; 14 Centre of Innovation in Interprofessional Health EducationUniversity of California, San FranciscoSan Francisco, CAUnited States; 15 Sunnybrook Health Sciences CentreDepartment of MedicineToronto, ONCanada; 16 Centre for Patient SafetyUniversity of TorontoToronto, ONCanada; 17 Centre for Interprofessional EducationUniversity of TorontoToronto, ONCanada

**Keywords:** hospital care communication, technology, knowledge transfer, interprofessional collaboration

## Abstract

**Background:**

Communication and collaboration failures can have negative impacts on the efficiency of both individual clinicians and health care system delivery as well as on the quality of patient care. Recognizing the problems associated with clinical and collaboration communication, health care professionals and organizations alike have begun to look at alternative communication technologies to address some of these inefficiencies and to improve interprofessional collaboration.

**Objective:**

To develop recommendations that assist health care organizations in improving communication and collaboration in order to develop effective methods for evaluation.

**Methods:**

An interprofessional meeting was held in a large urban city in Canada with 19 nationally and internationally renowned experts to discuss suitable recommendations for an ideal communication and collaboration system as well as a research framework for general internal medicine (GIM) environments.

**Results:**

In designing an ideal GIM communication and collaboration system, attendees believed that the new system should possess attributes that aim to: a) improve workflow through prioritization of information and detection of individuals’ contextual situations; b) promote stronger interprofessional relationships with adequate exchange of information; c) enhance patient-centered care by allowing greater patient autonomy over their health care information; d) enable interoperability and scalability between and within institutions; and e) function across different platforms. In terms of evaluating the effects of technology in GIM settings, participants championed the use of rigorous scientific methods that span multiple perspectives and disciplines. Specifically, participants recommended that consistent measures and definitions need to be established so that these impacts can be examined across individual, group, and organizational levels.

**Conclusions:**

Discussions from our meeting demonstrated the complexities of technological implementations in GIM settings. Recommendations on the design principles and research paradigms for an improved communication system are described.

## Introduction

Interprofessional communication between clinicians has traditionally relied on numeric paging systems that are riddled with numerous problems [1-4]. These include difficulty in identifying and contacting the right clinician, limited capability as a one-way receiver of information, and frequent interruptions. These may contribute to medical error and often result in increased frustration amongst clinicians [5,6]. There is a significant impact—poor communication practices with the resulting breakdowns on health care delivery. In a review of 14,000 hospital admissions, poor communication and collaboration practices were identified as the most common cause of preventable clinical errors [7]. Communication failures were also rated as one of the top most preventable causes for all known clinical errors [8]. Communication inefficiencies that resulted in wasted time for clinicians and increased length of stay for patients could cost North American hospitals as much as 12 billion dollars per year [9].

Recognizing the costs and problems associated with ineffective clinical communication, health care organizations have begun to look at emerging communication technologies to address some of these inefficiencies [10-12]. This coincides with an increased uptake of new mobile communications devices by clinicians, with an estimated 81% of North American physicians who currently own or use smartphone technology [13]. New communication technology that combines mobile phone, text, and email functions has the potential to improve clinical communication. Various hospitals have attempted different technological solutions to enhance their communication processes. These solutions have included the use of wireless email, two-way alphanumeric paging, smartphone communication, and a web-based communication tool that queues non-urgent messages [14]. 

Yet despite the increased adoption of communication technologies in health care, there is very little research that evaluates the effectiveness of these information and communication systems. Furthermore, rapidly changing technology is making evaluations and interpretations of these implementations challenging. Recent systematic reviews of smartphones concluded that there is limited evidence demonstrating that the use of technology leads to direct improvements in either clinician efficiency or patient care [14,15]. Consequently, many questions remain on the best evidence-based practices and strategies for these communication technologies.

Recognizing these gaps, an interprofessional meeting was organized to bring a diverse group of leading experts and stakeholders together to discuss effective ways to design and evaluate communication systems that can enhance clinical communication processes in health care organizations. Funded by the Canadian Institutes of Health Research (CIHR), the key objectives of the meeting were to 1) develop principles of an effective communication system, 2) identify key research priorities and paradigms in General Internal Medicine (GIM)–related communication areas, and 3) propose methods in which technological changes can be both implemented and evaluated successfully.

## Methods

### Workshop

An Interprofessional Communication and Collaboration Meeting was held in Toronto, Canada, on April 29, 2011, to discuss solutions that address the rapidly changing demands and needs of hospital communication and collaboration. Attendees came from different clinical and academic backgrounds to ensure that heterogeneous viewpoints were represented on a wide range of topics relating to interprofessional collaboration and information and communication technologies (ICT). The meeting involved the following steps: 1) the identification of an array of experts who were invited to participate from the fields of clinical communication and health care technologies. These experts were identified through literature reviews and professional networks and represented a diversity of professions that included medicine, nursing, pharmacy, academic research, health informatics, engineers, and hospital administration; 2) attendees were asked to provide position statements on the design and research models of an interdisciplinary team communication system, which were compiled and circulated among the group for review prior to the meeting; 3) the meeting began with presentations delivered by three of the invited experts who described their current research work and ICT implementations conducted at their respective hospitals and/or institutions; 4) based on their area(s) of expertise, participants were subsequently assigned to one of the two working groups: A) Design and Implementation or B) Research and Evaluation; 5) the working groups were co-facilitated by the invited experts who worked through previously formulated case studies and key questions (see Appendix 1) to develop recommendations for their respective domains; 6) the recommendations were presented to the larger group, which concluded with roundtable discussions among the attendees on the themes and issues raised during the sessions; 7) written field notes of the discussions were recorded by note-takers and subsequently transcribed into raw Microsoft Word documents after the meeting; 8) using an inductive thematic analysis, recommendations and discussion notes were then analyzed, summarized, and drafted for an initial report by two members of the research team (RW and VL); and 9) the report was sent to all participants for content validation. 

### Workshop Participants

The key issues related to clinical interprofessional communication and collaboration transcended the traditional disciplinary boundaries and demanded a broad range of interests and areas of expertise. These professional groups included medicine, nursing, pharmacy, academic research, health informatics, engineering, and hospital administration. Experts were identified through published literature as well as recommendations from professional networks. The invitees were selected by reviewing their experience and knowledge in various domains, publication records, and participation in initiatives or projects related to clinical interprofessional communication or ICT. Despite the efforts to have geographic, academic, and clinical diversity among the meeting invitees, many of the identified experts came from a few regions and organizations where active work in the field was being undertaken. To facilitate the process of inviting national and international experts, we made every effort to ensure out-of-town participants were able to attend in person or via videoconferencing (Skype). The majority of the invitees were able to attend the meeting in person with the exception of one attendee who participated via Skype.

### Definitions

Interprofessional communication was defined as information exchanges of patient-related issues between different care providers and professions. These included face to face, verbal, and text messages, and both scheduled communications such as interprofessional rounds and “as needed” or “ad-hoc” communications.

Interprofessional collaboration was defined as different professions working together as a team toward a common goal of providing optimal patient care using the skills/expertise of other professions.

## Results

Nineteen expert participants from Canada and the United States attended the Interprofessional Communication and Collaboration Meeting. This summary presents the major themes developed by the attendees in each session and concludes with issues and prospects for both the design/implementation and research communities (Table 1).

**Table 1:** Summary of major themes for design recommendations of an ideal communication system and recommendations for future research.

Theme	Subtheme
**Key Principles of the Ideal System**	
	Safety – The new communication system should help minimize communication errors and improve patient safety.
	Patient-Centered Focus – The system centers on the patient instead of specific providers, promoting the inclusiveness of all individuals and team integration.
	Cost – The cost of the existing communication inefficiencies outweighs the financial burden of implementing a new communication system.
**Design Recommendations**	
	Improve Workflow Through Contextual Awareness and Prioritization – Minimize interruptions by allowing message receivers to set their availability and prioritize messages by urgency.
	Promote Stronger Collaborative Relationships – Provide capability to communicate to more than one team members.
	Enhance Patient-Centered Care – Allow patients to be a part of the communications.
	Allow Interoperability and Scalability – Allow communication to clinicians providing care to patients, regardless of the institution with which they are affiliated.
	Support Multiple Technologies – Support different communication technologies such as pagers, cell phones, and different types of smartphones.
**Research Recommendations**	
	Considerations of the Contexts and Processes in Which Technology are Embedded – A broad approach looking at the processes and contexts in which these technologies are adopted including professional and organizational cultures.
	Need for Extensive Research Frameworks – It is important to examine the processes and how technology interacts from multiple perspectives, including 1) Education, 2) Clinical Practices, 3) Culture, 4) Interprofessional Collaboration and Communication, and 5) Organization of Care.
	Need for Multifaceted Outcome Measurements – Mixed methods consisting of qualitative and quantitative approaches should be used to obtain multiple data sources when evaluating complex interventions.

### Group A: Design and Implementation

A total of eight attendees were assigned to the Design and Implementation group. Participants of the group were tasked to identify important design principles for an improved communication and collaboration system that can be adopted in health care settings. The following key themes emerged from the discussions.

#### Themes

##### Key Fundamentals/Principles of the Ideal System

During the discussions, the group agreed that there should be key principles to guide in the design of the system such that the new communication system should produce positive or neutral impact at the very least. Specifically, the impact should affect the following areas:

###### Safety

The new communication system should help to minimize communication errors and improve patient safety. Participants noted that in the growing body of literature, patient safety has often been compromised by factors such as frequent interruptions in clinicians’ routines, which are exacerbated by the untimely delivery of messages. Thus, participants agreed that an ideal communication system should both minimize the occurrences of adverse events and improve patient safety.

###### Patient-Centered Focus

The interprofessional team should consider using one communication system to collaborate on the care of patients, as opposed to separate systems that merely serve the needs of different professions. Participants recognized that communication inefficiencies could create unnecessary patient inconvenience. Thus, there is a necessity for a system that could a) coordinate and manage all parts of the communication process, b) accomplish a defined set of goals that streamline workflow, and c) maximize practice efficiency and productivity amongst clinicians. Consequently, participants believed that the optimal model should encompass solutions that address these gaps of health care communication by promoting a) inclusiveness of all individuals and team integration, b) standardization the language used between team members, and c) timeliness and accuracy of information so that high-quality patient care can be delivered.

###### Cost

The cost of the existing communication inefficiencies outweighs the financial burden of implementing a new communication system. Participants acknowledged that strategic and operational considerations also need to be included in the long-term decision-making plans and sustainability of the health care systems. Thus, an important consideration for analysis is to understand the potential value, opportunities, and cost associated with the modifications or changes in the use of clinical communication resources. Subsequently, the incremental improvements in the new clinical communication system should have major positive impacts on overall health care expenditure, which should outweigh the financial cost of the new system. 

##### Design Recommendations

From the discussions, the following key design recommendations emerged as important features to a successful clinical communication system:

###### Improve Workflow Through Contextual Awareness and Prioritization

Participants observed that existing disruptions in communication and information transfer often create unnecessary inefficiencies in clinical workflow. Thus, one of the key goals of the ideal communication system should aim to improve clinical workflow whereby only essential communication interrupts clinicians. This could be accomplished through appropriate prioritization. However, it was acknowledged that the concept of urgency may vary depending on the perspective of the sender and the receiver. That is, a message deemed to be urgent by the sender may not be considered urgent by the receiver (and vice versa). Furthermore, although different clinicians may agree on the categorization of messaging, their perception of appropriate response time may differ. For example, although medical and nursing staff may agree that a meeting with the patient’s family members for an update is “non-urgent”, they may disagree as to how long the family should wait to speak to the physician. This discordance can escalate messaging frequency and in some cases lead to conflict. Some attendees proposed adopting practices such as standardizing a set of guidelines that define urgent and non-urgent issues. Others advocated that the system should provide clinicians the flexibility to designate the urgency of the message and the status of receiver. One possibility is to design a system that allows clinicians to indicate their situation and availability (ie, “context awareness”). For example, the system should enable clinicians from a range of professions to indicate their locations (eg, in an isolation room, in a teaching session, in the operating room) as well as their ability to respond to messages (eg, available, performing a procedure—do not interrupt, in a critical family meeting—do not interrupt). In the absence of a response from the original receiver, the system should have an algorithm to help escalate the sender’s messages to the next level and provide two-way feedback loops to both the sender and the receiver that the message and information has been escalated and dealt with.

###### Promote Stronger Collaborative Relationships

The second attribute of the ideal communication system should include features that promote teamwork and create stronger collaborative relationships. One of the biggest barriers to collaboration and cohesion involves misunderstandings and frustrations over discrepancies in the flow of information between clinicians. Often, clinicians face challenges identifying members of the patient’s care team resulting in information being transmitted between single individuals that excluded other GIM staff. Thus, another key aspect of the ideal system is to ensure an accurate list of the different clinicians caring for the patient that is integrated with the necessary communication channels. This would make it easy for all clinicians on the team to be included in the conversations by being updated and kept in the loop on the information they need. Moreover, in the area of message distribution, the system should have functionalities that allow mass broadcasting to all members of the clinical teams but also “tiering” for subgroups to receive specific targeted messages. Nonetheless, despite the support for new technological implementations and functionalities, caution should be taken to ensure that the new technology does not replace face-to-face interaction but rather augment it in ways that support team collaboration.

###### Enhance Patient-Centered Care

Participants agreed that while the system is designed for use mainly among clinicians, it should be one that promotes patient-centered communication focusing on unified communication strategies to connect patients and providers. Drawing from the perspective of the social networking model that looks at how relationships between individuals are connected between one another, attendees advocated that the patient should also be allowed to partake in the communication dialogue. At a minimum, the ideal communication system should keep patients informed on who is caring for them and be updated on the status of their care or treatment plans. Furthermore, attendees believed that patients should have a voice in how they would like to share their health care information. One possible way identified during the discussions was to design a communication system that either allows patients, family members, and/or physicians to determine the level of privacy and security in the flow of the patient health information and records. Thus, a patient-centric approach with the flexibility to designate user views and preferences should be considered. Nevertheless, these technological components should also be grounded in the principles of equality such that the system is highly usable by patients and their family members and is accessible regardless of age, culture, or ethnicity.

###### Allow Interoperability and Scalability

Attendees agreed that interoperability between institutions and health settings should be another important feature of the ideal system. Since many patients have multiple chronic diseases, they have multiple care providers distributed throughout the institutions and health settings. Specifically, the ideal system should establish common and understandable professional and interprofessional language and platforms that meet both clinicians’ and stakeholders’ needs. Additionally, the new system should be cost-effective and scalable to different hospital sizes, policies, and requirements. Moreover, the group agreed that user guidance and education should be provided alongside communication system implementation and knowledge transfer phases to encourage everyone to share the same goals and objectives.

###### Support Multiple Technologies

To date, industry solutions have focused on either expensive proprietary systems or the deployment of business/consumer-level communication devices and platforms in the health care marketplace. The lack of open and common standards is a significant barrier to application and device development for health care mobility at the point of care. Thus, considering the rapid advancement and evolution of new technology, the ideal system should be “device agnostic”. That is to say, the system should have the capabilities to accommodate different mediums of communication technologies that leverage diverse platforms and technologies rather than being constrained to one specific channel or platform. Nonetheless, the group also acknowledged the challenges and complexities of managing different communication mediums. Specifically, having multiple channels of communication (paging, text messaging, calling, or others) but without having a common understanding when it is appropriate to use each channels of communication, considerable confusion may be created among users. This could result in situations of delay and misunderstanding that could be worse than the traditional, simpler communication systems.

### Group B: Research and Evaluation

A total of 11 attendees were assigned to the Research and Evaluation group. Participants of the group were tasked with identifying the current research gaps and approaches in evaluating the effects and impact of a new communication system implementation. Specifically, participants were asked to consider the types of frameworks, methodologies, and theories that ought to be adopted when conducting evaluation research in the area of clinical communication and technology. The following themes emerged:

#### Themes

##### Research Recommendations

###### Considerations of the Contexts and Processes in Which Technology are Embedded

Participants recognized that current assessments and perspectives of health care communication are often fragmented. Existing studies often focus on technology and its direct impact alone, which is inadequate and limiting in understanding the complexities of communication in health care settings. Instead, participants advocated a broader approach of looking at the *processes *and *contexts *to which these technologies are adopted. Specifically, research on health care communication domains—such as interprofessional collaboration and ICT—need to consider other factors such as professional and organizational culture(s) and socialization processes, which are often intertwined. Experts argued that new technology interventions and designs should consider challenging the traditional workflow.

Attendees also recognized that when organizations are transitioning between systems, it is important to be aware that certain information or collaborative opportunities may be lost in the adoption of the new technology. Thus, it is important for researchers to consider the roles of technology and its processes at the organizational level by exploring interactions that occur between institutional cultures and technology. In particular, participants believed that the existence of different patterns of communication—brought about by the cultural aspects of professional tensions and hierarchies—may be the critical pieces to improving communication and collaboration.

###### Need for Extensive Research Frameworks

Participants agreed that existing research frameworks have been successful at tracking metrics such as monitoring patient outcomes that are often found in quality improvement interventions. Although these quality improvement studies may have provided meaningful knowledge on how to enhance the quality and safety of care, these studies often lacked the scholarly conceptualizations, as seen in the dearth of social and organizational level theories in explaining the phenomena. Experts acknowledged that the focus on technology usage is only one piece to understanding the dynamics of how information and communication technologies impact the field of interprofessional collaboration. Additionally, evaluators of health information technology should also assess the impacts of how these interventions affect different levels of the system and organization. Specifically, experts identified the following issues that researchers need to consider. These include examining the processes and how technology interacts with 1) Education, 2) Clinical Practices, 3) Culture, 4) Interprofessional Collaboration and Communication, and 5) Organization of Care.

###### Need for Multifaceted Outcome Measurements

Experts also recognized the challenges of identifying the appropriate measures and their assessments in the research models. Questions were raised over the types and definitions of outcomes and how to measure them. In the current literature, participants noted that different definitions and benchmarks were often used to measure similar concepts or outcomes, which are problematic for the researchers. For example, the definition of communication failures could be measured as either a) disruptions to the flow of information, b) frequency of communication events, or c) simple references and classifications of communication failures. Thus, suggestions were made that championed evaluation research to apply rigorous scientific approaches and designs.

Yet, the types of methods used to investigate these multifaceted and complex processes can be problematic and difficult for assessments. In particular, it was noted that complex interventions do not only affect one singular outcome but rather, produce multiple consequences on diverse areas including effects on patient safety through the adverse events, interprofessional relationships, and efficiencies of individuals, teams, and organizations. Thus, considering the complexities of the inherent processes, it was recommended that mixed methods consisting of qualitative and quantitative approaches be used to obtain multiple data sources when evaluating complex interventions. 

## Discussion

Overall, the discussion points raised in our meeting captured the current concerns raised by many implementers and researchers across various fields, disciplines, and professions. Participants acknowledged that there are essential uses for information and communication technologies in the clinical settings. Yet the creation of the ideal clinical communication will require integration of the design and research paradigms.

From the design and implementation perspective, discussions among the meeting’s participants revealed that the ideal health care communication system should be one that allows the large volume of patient information to be conveyed and shared at the *appropriate *time to the *right *person. The optimal communication design should be one that allows clinicians to choose *when *and *what *types of information they wish to receive through these communications. Participants also brought up the importance of designing a health care communication system that focuses on patient-centered care. Consequently, a communication system should allow patients to have greater autonomy in managing their health information and create more equality between patients and clinicians. Finally, the system should also be interoperable and scalable to different institutions that allow ease of knowledge transfer.

From the research and evaluation perspective, participants acknowledged that perspectives traditionally used to examine the role of technology in health care communication have been fragmented with researchers working in separate silos. Thus, new forms of integrative thinking and theorizing that span multiple disciplines and fields (eg, social sciences, quality and safety, and information technology) should be considered. Research priorities should consider the impact of communication technology on health care by examining it across different levels and units of analyses ranging from the organizational level (eg, organizational culture and hierarchies), to the team level (eg, workgroups and clinical practices), and finally at the individual level (eg, the clinician’s workflow). While the exploration of health care technology from different perspectives is an important step, the tools used to map the paths are equally as important. In order to illuminate insights into the complex and multifaceted processes that exist at multiple levels, participants advocated that research should explore novel methodologies and define theoretical frameworks as ways to further our understanding of clinical communication activities.

Looking from the lenses of interprofessional communication and collaboration, the rising trends in our aging populations and the emergence of chronic diseases also meant that an increasing number of these patients with chronic and complex conditions are being admitted into our hospitals today. There is a call for a communication system that promotes team cohesion and collaboration as hospitals further adopt an interprofessional approach involving different health care practitioners working in different temporal and spatial zones [16,17]. Thus, a strong communication and collaboration structure should be one that not only keeps all clinicians caring for the patient adequately informed but also one that could establish and standardize a common interprofessional language among diverse clinicians [18,19]. Additionally, providing adequate education and gaining buy-in from stakeholders and end users will be critical success factors for implementing this sophisticated technology in the health care system.

To the best of our knowledge, this is the first meeting with this agenda that has been previously organized. Thus, our meeting serves to expand the knowledge base in the fields of information and communication technologies as well as interprofessional collaboration. Through this meeting, we were able to bring different professionals, key stakeholders, and leading experts in the field of health care communication into one room to discuss how technology can serve the needs of today’s health care settings and determine ways to evaluate these implementations. We were able to identify the attributes of what the ideal communication system should entail and determine ways in which we can garner interest and partnerships to help develop and implement a sophisticated system successfully—one that not only improves efficiency at the system level but also promotes quality interprofessional collaboration and patient care. Moreover, we have identified the limitations of current knowledge and existing research gaps and proposed recommendations of what health care communication researchers can do to bridge those gaps. Thus, beyond the identification of existing issues, we were able to brainstorm recommendations that health care systems and researchers could apply in their organizations and fields of study alike.

A limitation of the meeting is that physicians who attended the workshop were primarily from the General Internal Medicine specialty who may be accustomed to particular communication cultures and patterns. Another limitation is that the meeting was hosted for only half a day, which may have constrained participants from delving further in their discussions or brainstorm more solutions and recommendations. Nonetheless, all the workshop participants felt that they were given sufficient time to express their opinions and believed the half-day workshop had achieved the intended goals and purposes. Also, given that the aim was exploratory and geared toward hypothesis generation, we believe that the findings provided us with sufficient grounding to make recommendations that will guide future work. While we had excellent interprofessional representation, representation by patients and family members may have provided improved understanding of their roles and on information governance and confidentiality. Finally, as communication systems become more advanced, information contained within these systems may overlap information with an electronic patient record. The meeting did not explore this issue and how this overlap should be managed but should be considered in the future.

### Conclusion

While there is a push to adopt technology as the solution to fix specific health care communication problems, collaboration between clinicians is complex, and answers to overcoming health care communication challenges extend beyond the selection of the latest technology. Health care institutions need to consider their strategies and examine how to effectively facilitate and integrate communication technology with different components of the overall system and organization. Caution is required to ensure that technological changes and implementations are adopted carefully to minimize the failures and unintended consequences by considering how interdependencies among multiple parts of the system — people, processes, policies, cultures, and physical infrastructure — influence health care communication and collaboration outcomes.

## Multimedia Appendix 1

Guiding questions and materials used in the Interprofessional Communication and Collaboration Meeting.
