# Supplementary material for: Improving Hospital Care and Collaborative Communications for the 21st Century: Key Recommendations for General Internal Medicine
Source: Interact J Med Res. 2012 Sep 24;1(2):e9. doi: 10.2196/ijmr.2022 (PMC3626135; doi:10.2196/ijmr.2022)
Supplement: Supplementary file 1 [file ijmr_v1i2e9_app1.pdf]

Appendix A: Guiding Questions and Materials used in the Interprofessional Communication and Collaboration Meeting

Group A: Design and Implementation

Group Activities	Key Considerations
**Breakout Session 1**	What are the important principles for an improved communication / collaboration system?<br>Consider from all perspective including front line clinicians, administrators, and patients.<br>*Please rank design principles.*
**Breakout Session 2**	Based on the principles, please describe what the design of an optimal communication/collaboration system should look like.<br>Please run through scenario A. Supplement with further scenario details to illustrate important aspects of the design.<br>How can we make the development of this system happen?<br>Consider what should be done to raise the interest?<br>Consider who should be our partners to build it.

Case Study

Instructions: Please consider communication from the perspectives of the patient, resident, nurses, and other clinicians. Please consider not just channels of communication but also how people determine *who* to call.

Patient A on ward is having new severe abdominal pain 8/10 for the last 2 hours. Vitals are stable. **Nurse B** needs to communicate urgently with most responsible resident. Most responsible **resident C** is on another ward assessing **patient D** in isolation. **Resident C** will indicate they will be up in about 15 minutes to assess.

Resident C finishes assessment of **Patient D** who was admitted with pneumonia, heart failure and dementia. Family wants to bring patient home today even though she is not at her baseline but they want to respect patient's wishes to go home. The planned discharge date discussed in morning rounds had been at least 3 days from today. Patient and family

needs to be seen by *pharmacy, CCAC (community nursing care coordinator), social worker, physiotherapy and occupational therapy* to have a safe discharge. Please indicate how the system would facilitate this communication and discharge.

Resident C then goes into a critical family meeting to break bad news about a new cancer diagnosis with a patient and his family. Five minutes later, **Patient E** then complains of a new episode of angina and dyspnea. He is assessed by **Nurse F** who has found him to be tachycardic but otherwise he has stable vitals. Please indicate how this communication will be resolved.

Group B: Research and Evaluation

Group Activities	Key Considerations
**Breakout Session 1**	- Please identify the current research gaps in designing an improved communication and collaboration system.<br>- Consider whether we asking the correct question.<br>- Is there a framework we should be using?<br>- What methodologies should we be using?<br>- Which theories of communication would further inform this work?<br>- *Please rank research gaps in terms of impact and difficulty.*
**Breakout Session 2**	- Assume an improved clinical communication and collaboration system will be implemented.<br>- Please describe how to evaluate the improved system. Possible outcomes could include on quality of care, patient safety, costs, workload.<br>- What are our next steps to lead research internationally?<br>- Describe 3 projects and possible funding sources
